# Benzimidazole derivatives endowed with potent antileishmanial activity

**DOI:** 10.1080/14756366.2017.1410480

**Published:** 2017-12-13

**Authors:** Michele Tonelli, Elena Gabriele, Francesca Piazza, Nicoletta Basilico, Silvia Parapini, Bruno Tasso, Roberta Loddo, Fabio Sparatore, Anna Sparatore

**Affiliations:** a Dipartimento di Farmacia, Università di Genova, Genova, Italy;; b Dipartimento di Scienze Farmaceutiche, Università degli Studi di Milano, Milano, Italy;; c Dipartimento di Scienze Biomediche Chirurgiche e Odontoiatriche, Università degli Studi di Milano, Milano, Italy;; d Dipartimento di Scienze Farmacologiche e Biomolecolari, Università degli Studi di Milano, Milano, Italy;; e Dipartimento di Scienze e Tecnologie Biomediche, Università di Cagliari, Cittadella Universitaria, Monserrato, Italy

**Keywords:** *Leishmania tropica* and *infantum*, promastigotes, anti-leishmania agents, alkyl benzimidazolium salts, 2-benzyl-1-lupinylbenzimidazole derivatives

## Abstract

Two sets of benzimidazole derivatives were synthesised and tested *in vitro* for activity against promastigotes of *Leishmania tropica* and *L. infantum*. Most of the tested compounds resulted active against both *Leishmania* species, with IC_50_ values in the low micromolar/sub-micromolar range. Among the set of 2-(long chain)alkyl benzimidazoles, whose heterocyclic head was quaternised, compound **8** resulted about 100-/200-fold more potent than miltefosine, even if the selectivity index (SI) versus HMEC-1 cells was only moderately improved. In the set of 2-benzyl and 2-phenyl benzimidazoles, bearing a basic side chain in position 1, compound **28** (2-(4-chlorobenzyl)-1-lupinyl-5-trifluoromethylbenzimidazole) was 12-/7-fold more potent than miltefosine, but exhibited a further improved SI. Therefore, compounds **8** and **28** represent interesting hit compounds, susceptible of structural modification to improve their safety profiles.

## Graphical Abstract

**Figure:**
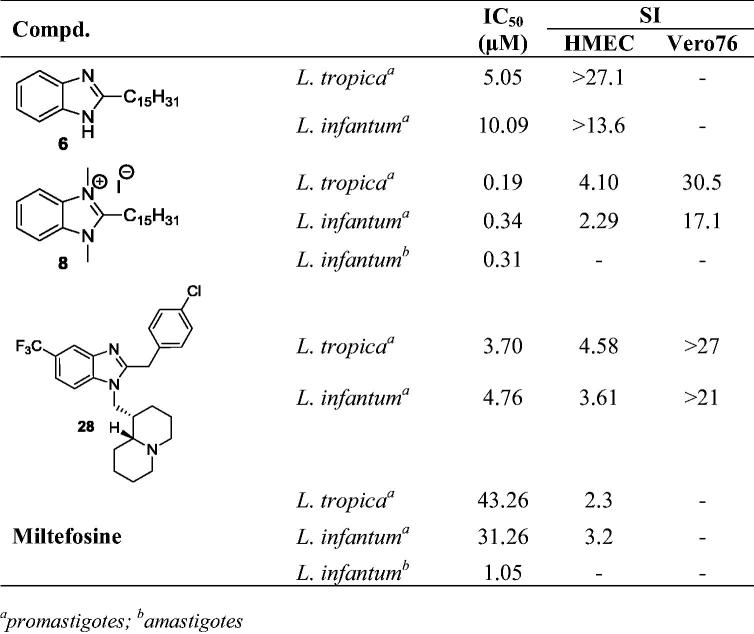

## Introduction

After malaria, leishmaniasis is the second most prevalent parasite infection worldwide for mortality in humans^1^. It is transmitted by the bite of a sand-fly infected by a flagellate protozoan of the genus *Leishmania*. Three different forms of the disease are described: visceral, cutaneous and muco-cutaneous leishmaniasis. The disease is endemic in many tropical and subtropical Countries, leading annually to an estimated 700,000–1 million new cases and 20,000–30,000 deaths^1^, mostly due to the visceral form caused by *Leishmania donovani*. The parasite exists in the ovoid non-flagellate form (amastigote) and in the flagellate promastigote, found in the sand-fly.

**Figure 1.**
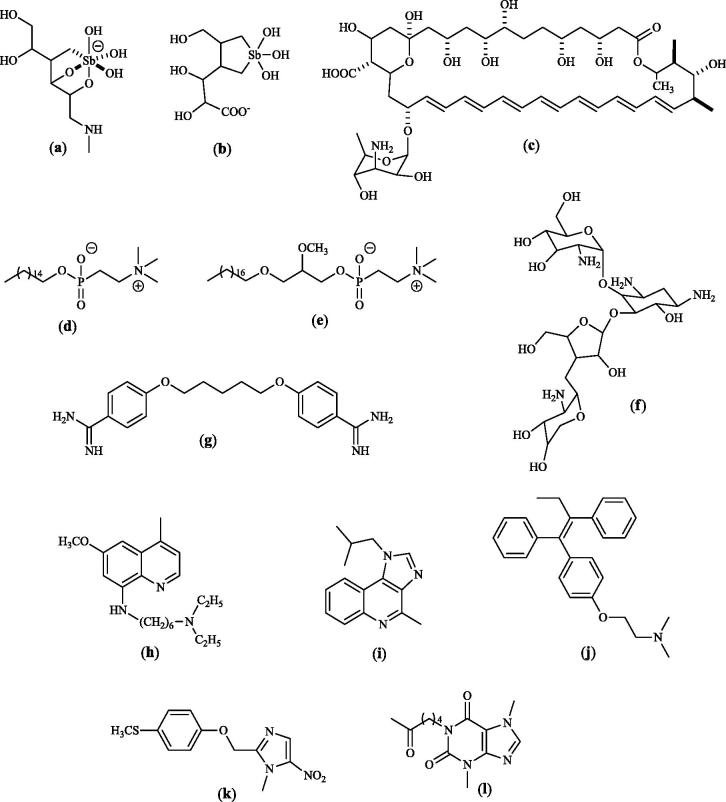
First and second line or synergistic agents to treat leishmaniasis: (a) meglumine antimoniate (predominant species in aqueous solution); (b) sodium stibogluconate (predominant species in aqueous solution); (c) amphotericin B; (d) miltefosine; (e) edelfosine; (f) paromomycin; (g) pentamidine; (h) sitamaquine; (i) imiquimod; (j) tamoxifene; (k) fexinidazole; (l) pentoxyphylline.

The therapy of leishmaniasis is still based on pentavalent antimonials (sodium stibogluconate and meglumine antimoniate) as first choice drug^2a^^,^^2b^, whereas amphotericin B, miltefosine, paromomycin and pentamidine are considered second-line drugs^3a–c^. Some other drugs as edelfosine, sitamaquine, fexinidazole, tamoxifene, imiquimod and pentoxyphylline are reported to give variable cure rates when used either alone or, better, in association with antimonials to overcome resistance^4^ (Figure 1).

All these drugs may cause several side effects and most of them are also expensive, and thus out of reach for the poor people living in tropical and sub-tropical countries, where the disease is endemic. The cited drugs exhibit very different chemical structures and hit a variety of biological targets, but in several cases the mechanism of action is still undefined or only partially known.

**Figure 2.**
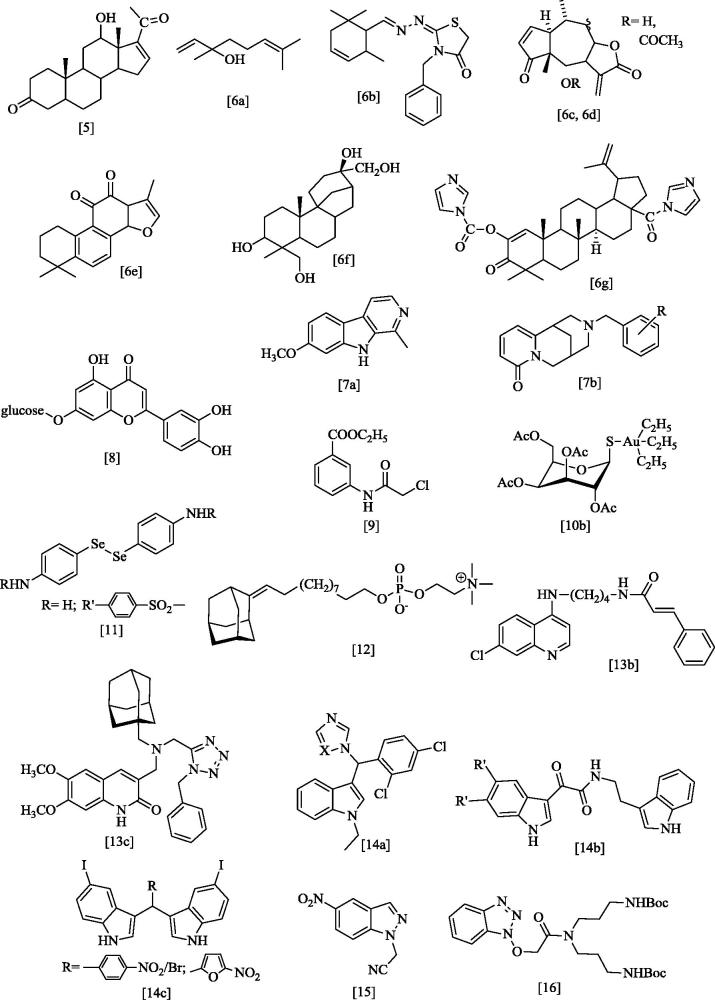
Examples of investigational anti-leishmanial agents^5–16^.

**Figure 3.**
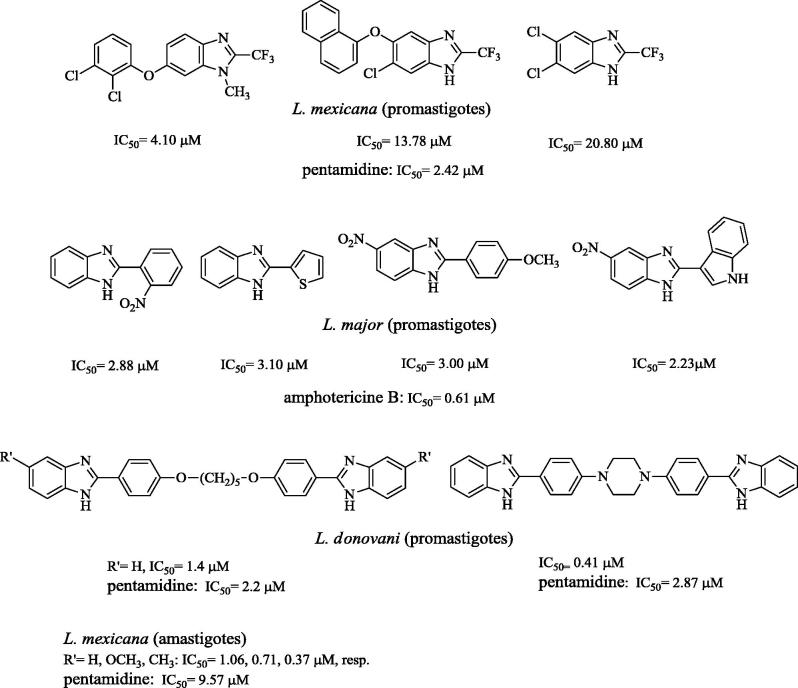
Benzimidazole derivatives previously tested as anti-leishmanial agents^17–20^.

To meet the need of novel more efficacious, safe and unexpensive drugs to treat leishmaniasis, a number of studies are on-going, exploring a wide chemical space from several classes of natural products^5^ and or their semi-synthetic derivatives (sterols^5^, mono-, sequi-, di- and tri-terpens^6^, alkaloids^7^, flavonoids^8^, etc.) to the most diversified synthetic compounds, from the simple chloroacetoanilides^9^, to organometallics^10a^ (as auranofin^10b^), aryldiselenides^11^, adamantylidene alkyl phosphocoline^12^ and a variety of heterocycles^13^, particularly indole^14^, indazole^15^, benzotriazole^16^ and benzimidazole^17–20^ derivatives. Examples of these compounds are depicted in Figures 2 and 3.

Among the benzazolic derivatives, an important position is held by the 2-trifluoromethyl-^17^ and 2-arylbenzimidazole^18^ derivatives that, besides activity versus several other protozoa, display antileishmanial action with potency in the low micromolar range. Interestingly, some bis-benzimidazoles^19^^,^^20^ exhibit sub-micromolar IC_50_, resulting 7- to 26-fold more potent than pentamidine.

**Figure 4.**
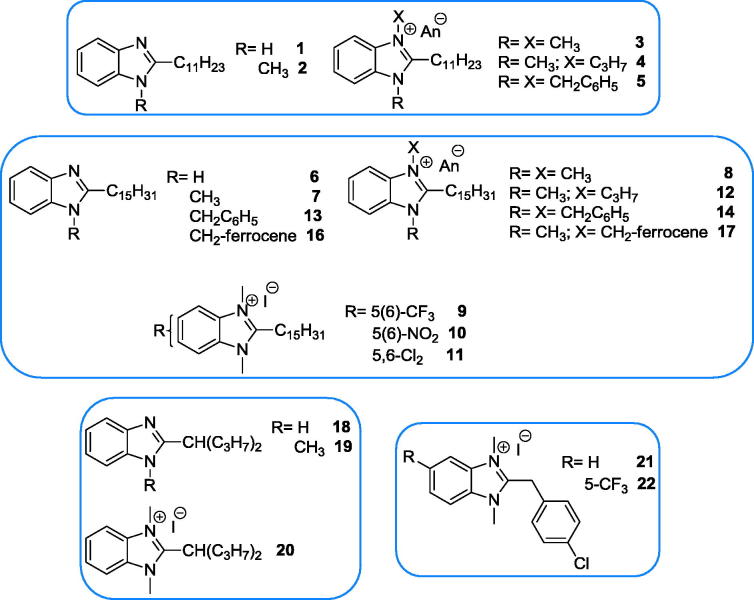
Investigated benzimidazole derivatives without basic side chain.

**Figure 5.**
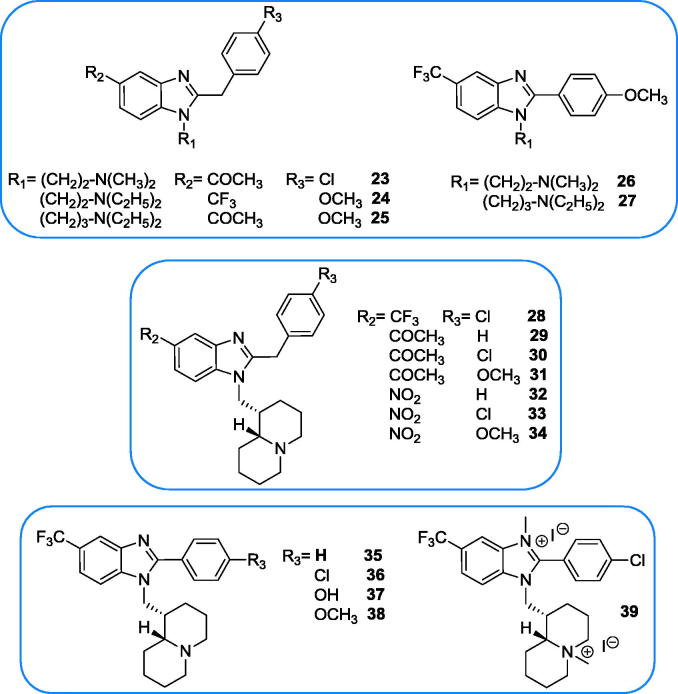
Investigated benzimidazole derivatives with basic side chain.

Since many years we are interested in the chemistry and biological properties of benzimidazole derivatives, pursuing varied pharmacological aims, from analgesic-anti-inflammatory action^21^, conditioned avoidance response (CAR) inhibition^22^, choleretic activity and gastric protection^23^, antiviral^24^ and antitumoral^25^ activities. In order to further explore the biocidal potential of benzimidazole derivatives, we deemed interesting to evaluate the antileishmanial activity of a set of 2-alkyl/2-benzyl benzimidazoles whose heterocyclic head was quaternised to mimic the ammonium head of miltefosine and edelfosine. Additionally, we selected, among our in house library of benzimidazoles, a second set of 2-arylbenzimidazoles 1-substituted with basic side chains that might be loosely related to sitamaquine. As the anti-leishmanial activity of sitamaquine analogues is mainly related to the length and structure of their basic side chains^4a^, in this subset of benzimidazoles a variety of basic chains, featured by different sizes, steric hindrance and lipophilicity, have been included. The bicyclic quinolizidine (lupinyl) moiety is of particular relevance, having been shown to produce analogous or superior activity against *Leishmania promastigotes* in comparison to sitamaquine^4c^ when replacing the diethylaminohexyl side chain of the latter (our unpublished results). On the whole 38 compounds (Figures 4 and 5) were tested against the promastigotes of *Leishmania tropica*, responsible for cutaneous leishmaniasis (CL), and 33 of them (depending on availability) were also tested against *L. infantum*, the causative agent of visceral leishmaniasis (VL). The two best compounds were also assayed against *L. infantum* amastigotes.

## Materials and methods

### General

Chemicals, solvents and reagents used for the syntheses were purchased from Sigma-Aldrich, Fluka or Alfa Aesar, and were used without any further purification unless otherwise stated. CC = flash column chromatography. Melting points (uncorrected) were determined with a Büchi apparatus. ^1^H NMR and ^13^C NMR spectra were recorded with a Varian Mercury 300VX or Varian Gemini-200 spectrometers in CDCl_3_ or acetone-d_6_; the chemical shifts were expressed in ppm (*δ*), coupling constants (*J*) in Hertz (Hz). High-resolution mass spectra (HRMS) were performed on a FT-Orbitrap mass spectrometer in positive electrospray ionisation (ESI). Elemental analyses were performed on a Carlo Erba EA-1110 CHNS instrument in the Microanalysis Laboratory of the Department of Pharmacy of Genoa University. Compounds were generally characterised by ^1^H and ^13^C NMR spectra and elemental analysis or HRMS; a few intermediates were characterised by elemental analysis and ^1^H NMR.

### General procedure for the synthesis of 1 H-benzimidazoles 1, 6

Benzene-1,2-diamine (500 mg, 4.62 mmol) and the appropriate acid (5.55 mmol) were stirred at 145 °C for 24 h under inert atmosphere. The resulting residue was purified by CC (silica gel; eluent as indicated for each compound). These compounds were already obtained through different procedure^26^^,^^27^.

*2-Undecyl-1 H-benzimidazole (****1****)*: CC (silica gel; cyclohexane/EtOAc; in gradient up to 92:8). The solid residue was rinsed with petroleum ether and the title compound was obtained as a white solid. Yield: 32%. m.p. 108.1–109.3 °C (lit.^26^, 107.5 °C). ^1^H NMR (300 MHz, CDCl_3_): 9.41 (s, 1H, NH). 7.56 (dd, 2H, *J* = 5.9 and 3.1 Hz), 7.22 (dd, 2H, *J* = 5.9 and 3.1 Hz), 2.94 (t, 2H, *J* = 7.7 Hz), 1.91–1.81 (m, 2H), 1.39–1.23 (m, 16H), 0.88 (t, 3H, *J* = 6.6 Hz). ^13^C NMR (50 MHz, CDCl_3_): 154.5, 137.3, 121.1, 113.5, 30.9, 28.6, 28.4, 28.35, 28.3, 27.4, 21.6, 13.1. Anal. Calcd for C_18_H_28_N_2_: C, 79.36; H, 10.36; N, 10.28. Found: C, 79.29; H, 10.53; N, 10.06.

*2-Pentadecyl-1 H-benzimidazole (****6****):* CC (silica gel; CH_2_Cl_2_/MeOH; in gradient up to 99.4:0.6). The solid residue was rinsed with ethyl ether and the title compound was obtained as a white solid. Yield: 63%. m.p. 88.8–94.2 °C (lit.^26^, 96.5–97; lit^27^, 98–100 °C). ^1^H NMR (300 MHz, CDCl_3_): 9.36 (s, 1H, NH), 7.56 (dd, 2H, *J* = 5.8 and 3.1 Hz), 7.22 (dd, 2H, *J* = 5.8 and 3.1 Hz), 2.29 (t, 2H, *J* = 7.7 Hz), 1.90–1.80 (m, 2H), 1.39–1.24 (m, 24H), 0.88 (t, 3H, *J* = 6.5 Hz). ^13^C NMR (50 MHz, CDCl_3_): 154.1, 136.5, 121.1, 113.6, 30.9, 28.6, 28.4, 28.3, 27.2, 21.6, 13.0. Anal. Calcd for C_22_H_36_N_2_: C, 80.43; H, 11.04; N, 8.53. Found: C, 80.50; H, 11.39; N, 8.42.

### General procedure for the synthesis of 2-alkyl-1-methyl-1 H-benzimidazoles (2, 7) and 2-alkyl-1,3-dimethyl-1 H-benzimidazol-3-ium iodides (3, 8)

To a solution of the appropriate 1*H*-benzimidazole (**1** or **6**, 0.37 mmol) in anhydrous THF (2 ml), K_2_CO_3_ (50.7 mg, 0.37 mmol) and methyl iodide (102 μL, 1.65 mmol) were added. The mixture was stirred at 40 °C for 76 h under inert atmosphere. After cooling at room temperature, inorganic salts were filtered and the solution was evaporated under reduced pressure. The resulting residue was treated with ethyl ether and rinsed with the same solvent giving compound **3** or **8** as a white-cream solid. The ethereal solution was then purified by CC (silica gel; eluent as indicated for each compound). Compounds **2**, **3** and **8** were already described in the literature^27–29^, obtained by different methods.

*1-Methyl-2-undecyl-1H-benzimidazole (****2****):* CC (CH_2_Cl_2_; isocratic). The solid residue was rinsed with ethyl ether and the final product was obtained as a white-cream solid. Yield: 45%. m.p. 40.8–43.4 °C (lit.^28^, yellow oil). ^1^H NMR (300 MHz, CDCl_3_): 7.75–7.72 (m, 1 H), 7.41–7.23 (m, 3H), 3.74 (s, 3H), 2.90 (t, 2H, *J* = 7.7 Hz), 1.87–1.84 (m, 2H), 1.45–0.85 (m, 19H); conforming to the previously described spectrum^28^.

*1,3-Dimethyl-2-undecyl-1 H-benzimidazol-3-ium iodide (****3****):* Yield: 22%. m.p. 157.3–161.3 °C (lit.^29^ 167–168 °C). ^1^H NMR (300 MHz, CDCl_3_): 7.68–7.62 (m, 4H), 4.11 (s, 6H), 3.55 (t, 2H, *J* = 7.2 Hz), 1.74–1.73 (m, 2H), 1.60–1.59 (m, 2H), 1.48–1.47 (m, 2H), 1.25–1.24 (m, 12H), 0.87–0.86 (m, 3H).^13^C NMR (75 MHz, CDCl_3_): 154.0, 131.4, 126.7, 112.5, 33.2, 31.6, 29.3, 29.1, 29.0, 28.9, 27.1, 26.1, 22.4, 13.8. HRMS (ESI) *m*/*z* Calcd for C_20_H_33_N_2_^+^ [M]^+^: 301.2638; found: 301.2637.

*1-Methyl-2-pentadecyl-1H-benzimidazole (****7****):* CC (CH_2_Cl_2_; isocratic). The solid residue was rinsed with petroleum ether and the final product was obtained as a white-cream solid. Yield: 17%. m.p. 64.2–65.6 °C. ^1^H NMR (300 MHz, CDCl_3_): 7.74–7.71 (m, 1H), 7.32–7.23 (m, 3H), 3.74 (s, 3H), 2.88 (t, 2H, *J* = 7.7 Hz), 1.92–1.82 (m, 2H), 1.45–1.25 (m, 24H), 0.88 (t, 3H, *J* = 6.6 Hz). Anal. Calcd for C_23_H_38_N_2_: C, 80.64; H, 11.18; N, 8.18. Found: C, 80.44; H, 11.20; N, 7.90.

*1,3-Dimethyl-2-pentadecyl-1 H-benzimidazol-3-ium iodide (****8****):* Yield: 43%. m.p. 169.0–172.0 °C (lit.^27^ 187–188 °C). ^1^H NMR (300 MHz, CDCl_3_): 7.72–7.70 (m, 2H), 7.69–7.59 (m, 2H), 4.11 (s, 6H), 3.55 (t, 2H, *J* = 7.7 Hz), 1.77–1.60 (m, 2H), 1.50–1.18 (m, 24H), 0.87 (t, 3H, *J* = 6.6 Hz).^13^C NMR (75 MHz, CDCl_3_): 154.2, 131.6, 126.9, 112.7, 33.3, 31.8, 29.6, 29.5, 29.3, 29.2, 27.3, 26.3, 22.6, 14.1. HRMS (ESI) *m*/*z* Calcd for C_24_H_41_N_2_^+^ [M]^+^: 357.3264; found: 357.3263.

### General procedure for the synthesis of 1-methyl-3-propyl-1 H-benzimidazol-3-ium iodides 4 and 12

To a solution of the appropriate 1-methyl-1*H*-benzimidazole (**2** or **7**, 0.16 mmol) in anhydrous THF (1 mL), 1-iodopropane (160 μL, 1.640 mmol) was added. The mixture was stirred at reflux for 24–60 h under nitrogen. After cooling at room temperature, ethyl ether was added to the reaction and the formed solid was then filtered and rinsed with the same solvent giving compound **4** or **12** as a white solid.

*1-Methyl-3-propyl-2-undecyl-1 H-benzimidazol-3-ium iodide (****4****):* Yield: 57%. m.p. 140.2–145.0 °C. ^1^H NMR (300 MHz, CDCl_3_): 7.71–7.61 (m, 4H), 4.40 (t, 2H, *J* = 7.2 Hz), 4.17 (s, 3H), 3.54 (t, 2H, *J* = 6.9 Hz), 2.05–2.03 (m, 2H), 1.74–1.73 (m, 2H), 1.55–1.52 (m, 3H), 1.25–1.24 (m, 13H), 1.10 (t, 3H, *J* = 7.4 Hz), 0.87–0.86 (m, 3H).^13^C NMR (75 MHz, CDCl_3_): 153.7, 131.8, 130.9, 126.8, 112.9, 112.7, 48.1, 33.7, 31.8, 29.5, 29.4, 29.3, 29.2, 29.1, 29.0, 27.1, 26.1, 23.1, 22.6, 14.1, 11.5. HRMS (ESI) m/z Calcd for C_22_H_37_N_2_^+^ [M]^+^: 329.2951; found: 329.2949.

*1-Methyl-2-pentadecyl-3-propyl-1H-benzimidazol-3-ium iodide (****12****):* Yield: 28%. m.p. 144.7–147.4 °C. ^1^H NMR (300 MHz, CDCl_3_): 7.73–7.59 (m, 4H), 4.40 (t, 2H, *J* = 7.5 Hz), 4.18 (s, 3H), 3.56 (t, 2H, *J* = 7.7 Hz), 2.08–2.01 (m, 2H), 1.77–1.70 (m, 2H), 1.59–1.53 (m, 3H), 1.40–1.25 (m, 21H), 1.12 (t, 3H, *J* = 7.4 Hz), 0.87 (t, 3H, *J* = 6.0 Hz). ^13^C NMR (75 MHz, CDCl_3_): 153.7, 131.8, 130.9, 126.8, 112.9, 112.7, 48.1, 33.7, 31.8, 29.6, 29.5, 29.4, 29.3, 29.1, 27.8, 26.1, 23.1, 22.6, 14.1, 11.5. HRMS (ESI) *m*/*z* Calcd for C_26_H_45_N_2_^+^ [M]^+^: 385.3577; found: 385.3580.

### General procedure for the synthesis of 2-alkyl-1,3-dibenzyl-1 H-benzimidazol-3-ium chlorides 5, 15 and 1-benzyl-2-pentadecyl-1 H-benzimidazole 13

To a mixture of K_2_CO_3_ (70 mg, 0.50 mmol) and the appropriate 1 H-benzo[d]imidazole (**1** or **6**, 0.30 mmol) in anhydrous THF (2.5 ml), benzyl chloride (183 μL, 1.52 mmol) was added, then stirred at reflux for 60 h under inert atmosphere. After cooling at room temperature, inorganic salts were filtered and the solution was evaporated under reduced pressure. The resulting residue was treated with THF and rinsed with the same solvent giving compound **5** or **15** as a white-cream solid. The solution was then purified by CC (silica gel; eluent as indicated for each compound).

*1,3-Dibenzyl-2-undecyl-1H-benzimidazol-3-ium chloride (****5****):* Yield: 77%. m.p. 223.3–225.3 °C. ^1^H NMR (300 MHz, CDCl_3_): 7.60–7.51 (m, 4H), 7.37–7.26 (m, 10H), 5.90 (s, 4H), 3.64 (t, 2H, *J* = 7.7 Hz), 1.20–0.99 (m, 18H), 0.86 (t, 3H, *J* = 6.7 Hz). ^13^C NMR (75 MHz, CDCl_3_): 155.9, 133.4, 131.7, 129.4, 128.8, 126.9, 126.7, 113.3, 49.8, 31.8, 29.6, 29.4, 29.3, 29.2, 29.1, 28.8, 27.3, 26.1, 22.6, 14.1. HRMS (ESI) *m*/*z* Calcd for C_32_H_41_N_2_^+^ [M]^+^: 453.3264; found: 453.3257.

*1-Benzyl-2-pentadecyl-1H-benzimidazole (****13****):* CC (CH_2_Cl_2_; isocratic). The solid residue was rinsed with cold MeOH and the final product was obtained as a white-cream solid. Yield: 17%. m.p. 60.7–61.8 °C. ^1^HNMR (300 MHz, CDCl_3_): 7.78–7.75 (d, 1H, *J* = 7.5 Hz), 7.30–7.18 (m, 6H), 7.05–7.03 (m, 2H), 5.34 (s, 2H), 2.82 (t, 2H, *J* = 7.6 Hz), 1.87–1.77 (m, 2H), 1.34–1.25 (m, 24H), 0.88 (t, 3H, *J* = 6.5 Hz). Anal. calcd for C_29_H_42_N_2_: C, 83.20; H, 10.11; N, 6.69. Found: C, 83.16; H, 10.41; N, 6.86.

*1,3-Dibenzyl-2-pentadecyl-1 H-benzimidazol-3-ium chloride (****15****):* CC (silica gel; CH_2_Cl_2_/MeOH; in gradient up to 99.5:0.5). The solid residue was rinsed with THF and the final product was obtained as a white-cream solid. Yield: 40%. m.p. 214.9–216.3 °C. ^1^H NMR (300 MHz, CDCl_3_): 7.56–7.54 (m, 4H), 7.36–7.29 (m, 10H), 5.90 (s, 4H), 3.67–3.65 (m, 2H), 1.25–0.87 (m, 29H). ^13^C NMR (75 MHz, CDCl_3_): 156.0, 133.3, 131.6, 129.4, 128.8, 126.9, 126.7, 113.2, 49.8, 31.8, 29.7, 29.6, 29.5, 29.4, 29.3, 29.0, 28.8, 27.3, 26.1, 22.6, 14.1. HRMS (ESI) *m*/*z* Calcd for C_36_H_49_N_2_^+^ [M]^+^: 509.3890; found: 509.3878.

### 3-Benzyl-1-methyl-2-pentadecyl-1 H-benzimidazol-3-ium chloride (14)

Benzyl chloride (168 μL, 1.43 mmol) was added to a solution of 1-methyl-2-pentadecyl-1*H*-benzimidazole (compound **7**, 0.15 mmol) in anhydrous THF (1 ml). The reaction was stirred at reflux for 80 h under inert atmosphere. After cooling at room temperature, the formed solid was filtered and rinsed first with THF and then with ethyl ether, providing compound **14** as a white solid. Yield: 18%. m.p. 215.7–219.3 °C. ^1^H NMR (300 MHz, CDCl_3_): 7.77–7.26 (m, 9H), 5.84 (s, 2H), 6.87 (s, 3H), 3.67 (s, 2H), 1.34–1.14 (m, 26H), 0.88 (t, 3H, *J* = 6.1 Hz). ^13^C NMR (75 MHz, CDCl_3_): 155.5, 133.5, 131.8, 131.5, 129.4, 128.9, 126.9, 126.8, 112.9, 112.8, 77.0, 49.7, 32.9, 31.9, 29.7, 29.6, 29.5, 29.4, 29.3, 29.2, 29.1, 27.2, 25.8, 22.7, 14.1. HRMS (ESI) *m*/*z* Calcd for C_30_H_45_N_2_^+^ [M]^+^: 433.3577; found: 433.3576.

### 1-(Ferrocenylmethyl)-2-pentadecyl-1 H-benzimidazole (16)

To a mixture of K_2_CO_3_ (63 mg, 0.45 mmol) and 2-pentadecyl-1*H*-benzimidazole (compound **6**, 0.30 mmol) in anhydrous THF/CH_3_CN (1:3.5 mL), ferrocenylmethyl trimethylammonium iodide (117 mg, 0.46 mmol) was added. The reaction was stirred at room temperature for 18 h under inert atmosphere. After the completion of reaction, the solution was evaporated under reduced pressure. The resulting residue was taken up with CH_2_Cl_2_ and washed several times with H_2_O. The organic layer was dried with anhydrous Na_2_SO_4_, filtered and evaporated to dryness to obtain a pale orange oil, which was purified by CC (silica gel; CH_2_Cl_2_; isocratic). The title compound was obtained as a pale yellow solid. Yield: 75%. m.p. 77.2–78.7 °C. ^1^H NMR (300 MHz, CDCl_3_): 7.79 (d, 1H, *J* = 6.3 Hz), 7.38 (s, 1H), 7.23–7.22 (m, 2H), 5.05 (s, 2H), 4.42–4.11 (m, 9H), 2.90 (t, 2H, *J* = 7.1 Hz), 1.89–1.88 (m, 2H), 1.64–1.25 (m, 24H), 0.88–0.85 (m, 3H). Hydrochloride: m.p. 149.8–150.2 °C. Anal. calcd for C_33_H_47_ClFeN_2_: C, 70.40; H, 8.41; N, 4.98. Found: C, 70.42; H, 8.91; N, 5.02.

### 1-(Ferrocenylmethyl)-3-methyl-2-pentadecyl-1 H-benzimidazol-3-ium iodide (17)

Methyl iodide (200 μL, 3.24 mmol) was added to a solution of 1-ferrocenyl-3-methyl-2-pentadecyl-1 *H*-benzo[d]imidazole (compound **16**, 0.09 mmol) in anhydrous ethyl ether (1.5 mL). The reaction was stirred at 40 °C for 80 h under inert atmosphere. After cooling at room temperature, the formed solid was filtered and rinsed with ethyl ether giving compound **17** as a white solid. Yield: 52%. m.p. 162.2–165.7 °C. ^1^H NMR (300 MHz, CDCl_3_): 7.74–7.73 (m, 1H), 7.60–7.59 (m, 3H), 5.55 (s, 2H), 4.36–4.21 (m, 9H), 4.05 (s, 3H), 3.53–3.52 (m, 2H), 1.52–1.51 (m, 4H), 1.26–1.24 (m, 22H), 0.88–0.87 (m, 3H). ^13^C NMR (75 MHz, CDCl_3_): 153.8, 131.6, 130.9, 126.7, 113.0, 112.6, 79.3, 69.4, 69.3, 47.1, 33.1, 31.9, 29.7, 29.6, 29.5, 29.3, 29.2, 27.5, 26.6, 22.6, 14.1. HRMS (ESI) *m*/*z* Calcd for C_34_H_49_N_2_Fe^+^ [M]^+^: 541.3240; found: 541.3234.

### 2-(Heptan-4-yl)-1 H-benzimidazole (18)

2-Propylpentanoyl chloride (338 mg, 2.08 mmol) was added at 0 °C to a solution of benzene-1,2-diamine (225 mg, 2.08 mmol) in anhydrous 1,4-dioxane (1 mL) and the reaction mixture was stirred at room temperature for 15 h under nitrogen. After that time, BF_3_^.^Et_2_O (263 μL) was added and the mixture was stirred at reflux for other 12 h. The solvent was then stripped off and the obtained residue was diluted with EtOAc, washed with a cold solution of 5% HCl and with 2 M NaOH. The organic layer was dried with anhydrous Na_2_SO_4_, filtered and evaporated to dryness to obtain a residue that was purified by CC (silica gel; CH_2_Cl_2_/cyclohexane; in gradient up to 80:20). The fractions containing the purified product were gathered up and rinsed with diethyl ether to provide a white solid. Yield: 18%. m.p. 224.7–225.8 °C. ^1^H NMR (300 MHz, acetone-d_6_): 7.49–7.46 (m, 2H), 7.39 (s, 1H), 7.13–7.11 (m, 2H), 3.03–2.95 (m, 1H), 1.91–1.81 (m, 2H), 1.79–1.64 (m, 2H), 1.33–1.20 (m, 4H), 0.89–0.84 (m, 6H). ^13^C NMR (50 MHz, CDCl_3_): 157.8, 136.8, 121.2, 113.6, 39.4, 36.1, 19.2, 12.9. Anal. calcd for C_14_H_20_N_2_: C, 77.73; H, 9.32; N, 12.95. Found: C, 77.71; H, 9.66; N, 12.83.

### General procedure for the synthesis of 2-(heptan-4-yl)-1-methyl-1 H-benzimidazole 19 and 1,3-dimethyl-2-(heptan-4-yl)-1 H-benzimidazol-3-ium iodide 20

To a mixture of K_2_CO_3_ (33.0 mg, 0.24 mmol) and 2-(heptan-4-yl)-1*H*-benzimidazole (compound **18**, 0.24 mmol) in anhydrous THF (1 mL), methyl iodide (814 μL, 13.14 mmol) was added. The reaction was stirred at 40 °C for 26 h under inert atmosphere. After cooling at room temperature, inorganic salts were filtered and the solution was evaporated under reduced pressure. The resulting residue was treated with ethyl ether and rinsed with the same solvent giving compound **20** as a white solid. The ethereal solution was then purified by CC (silica gel; eluent as indicated for each compound).

*2-(Heptan-4-yl)-1-methyl-1 H-benzimidazole (****19****):* CC (CH_2_Cl_2_; isocratic). The title compound was obtained as a pale grey oil. Yield: 47%. ^1^H NMR (300 MHz, CDCl_3_): 7.80–7.77 (m, 1H), 7.33–7.24 (m, 3H), 3.76 (s, 3H), 3.03–2.97 (m, 1H), 2.01–1.89 (m, 2H), 1.81–1.70 (m, 2H), 1.34–1.17 (m, 4H), 0.87 (t, 6H, *J* = 7.4 Hz). ^13^C NMR (50 MHz, CDCl_3_): 155.6, 130.7, 129.9, 125.4, 124.9, 114.9, 110.0, 36.6, 34.9, 30.5, 19.9, 12.7. Hydrochloride, m.p. 169.2–173.2 (EtOH/Et_2_O). Anal. calcd for C_15_H_23_ClN_2_: C 67.51, H 8.69, N 10.50, found: C 67.71, H 8.88, N 10.40.

*1,3-Dimethyl-2-(heptan-4-yl)-1 H-benzimidazol-3-ium iodide (****20****):* White powder. Yield: 25%. m.p. 192.2–192.9 °C. ^1^H NMR (300 MHz, CDCl_3_): 7.88–7.86 (m, 2H), 7.68–7.65 (m, 2H), 4.25 (m, 6H), 3.78–3.73 (m, 1H), 2.06–1.98 (m, 2H), 1.60–1.43 (m, 4H), 1.22–1.18 (m, 2H), 0.98–0.93 (t, 6H, *J* = 7.1 Hz). ^13^C NMR (75 MHz, CDCl_3_): 154.9, 133.1, 131.6, 127.4, 37.3, 34.5, 21.4, 13.9. HRMS (ESI) *m*/*z* Calcd for C_16_H_25_N_2_^+^ [M]^+^: 245.2012; found: 245.2011.

### General procedure for the synthesis of N-(2-aminophenyl)palmitamide derivatives 40–42

To a solution of the proper 4- or 4,5-substituted 1,2-phenylendiamine (2.5 mmol) in THF (8 mL) in presence of Hunig base (5 mmol), a solution of palmitoyl chloride (2.5 mmol) in 5 ml of THF was added dropwise. The mixture was reacted at r.t. for 24 h with stirring. After removing the solvent, the residue was taken up with water, alkalinised with 2 N NaOH and exhaustively extracted with CHCl_3_. The dried organic layer (Na_2_SO_4_) was concentrated to dryness leaving a residue that was thoroughly washed with dry Et_2_O/hexane (1:1).

*N-[2-Amino-4(5)-trifluoromethylphenyl]palmitamide (****40****):* White powder. Yield: 42%. m.p. 71–73.5 °C (hexane/Et_2_O an.). ^1^H NMR (200 MHz, CDCl_3_): 7.63 (s, 1H, NHCO, collapses with D_2_O), 7.39 (s, 1H), 7.26 (d, 1H, *J* = 8.8 Hz), 6.75 (d, 1H, *J* = 8.8 Hz), 3.92 (s, 2H, NH_2_, collapse with D_2_O), 2.41 (t, 2H, *J* = 7.8 Hz), 1.87–1.56 (m, 2H), 1.29 (pseudo s, 24H), 0.91 (t, 3H, *J* = 6.8 Hz). Anal. calcd for C_23_H_37_F_3_N_2_O: C, 66.64; H, 9.00; N, 6.76. Found: C, 66.72; H, 9.09; N, 6.85.

*N-[2-Amino-4(5)-nitrophenyl]palmitamide (****41****):* Yellowish powder. Yield: 29%. m.p. 142–143 °C (hexane/Et_2_O an.). ^1^H NMR (200 MHz, CDCl_3_): 8.06 (br. s, 1H and 1H, NHCO, collapses with D_2_O, superimposed), 7.42 (m, 1H), 6.82 (d, 1H, *J* = 8.8 Hz), 3.40 (s, 2H, NH_2_, collapse with D_2_O), 2.49 (pseudo s, 2H), 1.96–1.70 (m, 2H), 1.30 (pseudo s, 24 H), 0.92 (pseudo s, 3H). Anal. calcd for C_22_H_37_N_3_O_3_: C, 67.49; H, 9.53; N, 10.73. Found: C, 67.74; H, 9.57; N, 10.93.

*N-(2-Amino-4,5-dichlorophenyl)palmitamide (****42****):* White powder. Yield: 35%. m.p. 96–98 °C (hexane/Et_2_O an.). ^1^H NMR (200 MHz, CDCl_3_): 7.40 (s, 1H, NHCO, collapses with D_2_O), 7.36 (s, 1H), 6.95 (s, 1H), 3.38 (s, 2H, NH_2_, collapse with D_2_O), 2.42 (t, 2H, *J* = 7.8 Hz), 1.82–1.54 (m, 2H), 1.26 (pseudo s, 24H), 0.91 (t, 3H, *J* = 7.0 Hz). Anal. calcd for C_22_H_36_Cl_2_N_2_O: C, 63.60; H, 8.73; N, 6.74. Found: C, 63.51; H, 8.73; N, 7.00.

### General procedure for the synthesis of 2-pentadecyl-5/6-1H-benzimidazole derivatives 43–45

The N-(2-aminophenyl)palmitamides (0.50 mmol) in 4 N HCl (10 mL) were refluxed at 120 °C for 4 h. After cooling, the acidic solution was basified with 2 N NaOH and shaken with CH_2_Cl_2_. The organic layer was dried (Na_2_SO_4_) and evaporated to afford the benzimidazole that was thoroughly washed with dry Et_2_O/hexane (1:1). 2-Pentadecyl-5-trifluoromethyl-1*H*-benzimidazole was yield as an oil and was used as such for the preparation of the corresponding 1-methylbenzimidazole derivative.

*2-Pentadecyl-5-trifluoromethyl-1 H-benzimidazole (****43****):* Yield: 76%. Oil. ^1^H NMR (200 MHz, CDCl_3_): 9.24 (s, 1H, NH, collapses with D_2_O), 8.21 (s, 1H), 8.08 (d, 1H, *J*= 8.6 Hz), 7.80 (d, 1H, *J* = 8.6 Hz), 2.45 (t, 2H, *J* = 7.0 Hz), 1.84–1.1.59 (m, 2H), 1.29 (pseudo s, 24H), 0.91 (t, 3H, *J* = 6.8 Hz). Anal. calcd for C_23_H_35_F_3_N_2_: C, 69.67; H, 8.90; N, 7.06. Found: C, 69.45; H, 9.00; N, 8.75.

*5-Nitro-2-pentadecyl-1 H-benzimidazole (****44****):* Yield: 45%. m.p. 86–88 °C (hexane/Et_2_O an.). ^1^H NMR (200 MHz, CDCl_3_): 9.54 (s, 1H, NH, collapses with D_2_O), 8.52 (s, 1H), 8.21 (d, 1H, *J* = 9.0 Hz), 7.64 (d, 1H, *J* = 8.8 Hz), 3.04 (t, 2H, *J* = 8.0 Hz), 2.03–1.80 (m, 2H), 1.26 (pseudo s, 24H), 0.90 (t, 3H, *J* = 6.8 Hz). Anal. calcd for C_22_H_35_N_3_O_2_: C, 70.74; H, 9.44; N, 11.25. Found: C, 70.60; H, 9.59; N, 11.55.

*5,6-Dichloro-2-pentadecyl-1 H-benzimidazole (****45****):* Yield: 44%. m.p. 74–76 °C (hexane/Et_2_O an.). ^1^H NMR (200 MHz, CDCl_3_): 9.38 (s, 1H, NH, collapses with D_2_O), 7.60 (s, 1H), 7.26 (s, 1H), 3.01 (t, 2H, *J* = 8.0 Hz), 2.01–1.80 (m, 2H), 1.23 (pseudo s, 24H), 0.90 (t, 3H, *J* = 6.6 Hz). Anal. calcd for C_22_H_34_Cl_2_N_2_: C, 66.49; H, 8.62; N, 7.05. Found: C, 66.70; H, 8.95; N, 7.35.

### General procedure for the synthesis of N-methyl-1 H-benzimidazole derivatives 46–48, 50 and 51

In a sealed tube, to a solution of the proper benzimidazole (0.10 mmol) in 5 mL of THF were added, in the order, Cs_2_CO_3_ (0.30 mmol) and iodomethane (0.15 mmol). The mixture was heated at 60 °C for 6–8 h with stirring. The solvent was evaporated and the residue was taken up with water, alkalinised with 2 N NaOH and extracted with CH_2_Cl_2_. After drying, the solvent was removed obtaining an oily residue that was washed with hexane.

*N-Methyl-2-pentadecyl-5(6)-trifluoromethyl-1 H-benzimidazole (****46****):* White powder. Yield: 90%. m.p. 65.8–67.9 °C (hexane). ^1^H NMR (200 MHz, CDCl_3_): 8.03 (s, 1H), 7.81 (d, 1H, *J*= 9.6 Hz), 7.51 (d, 1H, *J* = 9.6 Hz), 3.79 (s, 3H, NCH_3_), 2.90 (t, 2H, *J* = 8.0 Hz), 2.01–1.80 (m, 2H), 1.28 (pseudo s, 24H), 0.89 (t, 3H, *J* = 6.4 Hz). Anal. calcd for C_24_H_37_F_3_N_2_: C, 70.21; H, 9.08; N, 6.82. Found: C, 69.72; H, 9.23; N, 6.00.

*N-Methyl-5(6)-nitro-2-pentadecyl-1 H-benzimidazole (****47****):* Yellowish powder. Yield: 45%. m.p. 69.7–71.4 °C (hexane). ^1^H NMR (200 MHz, CDCl_3_): 8.55 (s, 1H), 8.38 (d, 1H, *J*= 9.8 Hz), 7.68 (d, 1H, *J* = 9.8 Hz), 4.09 (s, 3H, NCH_3_), 3.28 (t, 2H, *J* = 7.8 Hz), 2.09–1.85 (m, 2H), 1.28 (pseudo s, 24H), 0.90 (t, 3H, *J* = 6.2 Hz). Anal. calcd for C_23_H_37_N_3_O_2_: C, 71.28; H, 9.62; N, 10.84. Found: C, 71.28; H, 9.67; N, 11.18.

*N-Methyl-5,6-dichloro-2-pentadecyl-1H-benzimidazole (****48****):* White powder. Yield: 42%. m.p. 64.8–67.3 °C (hexane). ^1^H NMR (200 MHz, CDCl_3_): 8.15 (s, 1H), 7.69 (s, 1H), 3.97 (s, 3H, NCH_3_), 3.28 (t, 2H, *J* = 8.4 Hz), 2.06–1.87 (m, 2H), 1.28 (pseudo s, 24H), 0.91 (t, 3H, *J* = 6.4 Hz). Anal. calcd for C_23_H_36_Cl_2_N_2_: C, 67.14; H, 8.82; N, 6.81. Found: C, 67.17; H, 8.80; N, 7.15.

*N-Methyl-2–(4-chlorobenzyl)-1 H-benzimidazole (****50****):* White powder. Yield: 23%. m.p. 117–119 °C (hexane) conforming to the literature^30^.

*N-Methyl-2-(4-chlorobenzyl)-5-trifluoromethyl-1 H-benzimidazole (****51****):* White powder. Yield: 100%. Oil. ^1^H NMR (200 MHz, CDCl_3_): 7.80–7.04 (m, 7H), 4.36 (s, 2H), 3.95 (s, 3H, NCH_3_). Anal. calcd for C_16_H_12_ClF_3_N_2_: C, 59.18; H, 3.72; N, 8.63. Found: C, 59.30; H, 3.65; N, 8.49.

### General procedure for the synthesis of benzimidazole quaternary ammonium salts 9–11, 21, 22 and 39

The suitable N-methylbenzimidazole derivative or N-lupinyl-5-trifluoromethyl-2-(4-chlorophenyl)benzimidazole (0.20 mmol) was reacted with iodomethane (0.5 mL, 8 mmol) at r.t. for 24 h with stirring. The reaction mixture was washed with dry Et_2_O affording the title quaternary ammonium salt.

*1,3-Dimethyl-2-pentadecyl-5-trifluoromethyl-1 H-benzimidazol-3-ium iodide (****9****):* Yield: 83%. m.p. 116–118 °C (Et_2_O an.). ^1^H NMR (200 MHz, CDCl_3_): 8.10–7.70 (m, 3H), 4.15 (s, 6H), 3.52 (t, 2H, *J* = 7.05 Hz), 1.93–1.64 (m, 2H), 1.57–0.80 (m, 27H). ^13^C NMR (50 MHz, CDCl_3_): 156.2, 132.7, 130.3, 122.7, 113.2, 109.6, 33.0, 32.6, 30.9, 28.6, 28.5, 28.4, 28.3, 28.1, 26.2, 26.0, 21.6, 13.1. Anal. calcd for C_25_H_40_F_3_IN_2_: C, 54.35; H, 7.30; N, 5.07. Found: C, 54.33; H, 6.92; N, 5.21.

*1,3-Dimethyl-5-nitro-2-pentadecyl-1 H-benzimidazol-3-ium iodide (****10****):* Yield: 51%. m.p. 154–156 °C (Et_2_O an.). ^1^H NMR (200 MHz, CDCl_3_): 8.63 (s, 1H), 8.29–8.17 (m, 1H), 7.88 (d, 1H, *J* = 8.10 Hz), 4.21 (s, 6H), 2.94 (t, 2H, *J* = 7.15 Hz), 2.00–0.80 (m, 29H). ^13^C NMR (50 MHz, CDCl_3_): 157.0, 133.0, 129.7, 117.0, 114.8, 107.7, 30.9, 29.2, 28.6, 28.4, 28.3, 26.7, 26.3, 21.7, 13.1; Anal. calcd for C_24_H_40_IN_3_O_2_: C, 54.44; H, 7.61; N, 7.94. Found: C, 54.37; H, 7.63; N, 7.98.

*5,6-Dichloro-1,3-dimethyl-2-pentadecyl-1 H-benzimidazol-3-ium iodide (****11****):* Yield: 100%. m.p. 200–203 °C (Et_2_O an.). ^1^H NMR (200 MHz, CDCl_3_): 7.96 (s, 2H), 4.14 (s, 6H), 3.51 (t, 2H, J = 7.15 Hz), 1.84–0.75 (m, 29H). ^13^C NMR (50 MHz, CDCl_3_): 155.5, 130.8, 129.7, 113.4, 32.4, 30.9, 28.6, 28.3, 26.2, 21.7, 13.1. Anal. calcd for C_24_H_39_Cl_2_IN_2_: C, 52.09; H, 7.10; N, 5.06. Found: C, 52.18; H, 7.17; N, 5.41.

*2-(4-Chlorobenzyl)-1,3-dimethyl-1 H-benzimidazol-3-ium iodide (****21****):* Yield: 35%. m.p. 94–98 °C (Et_2_O an.). ^1^H NMR (200 MHz, CDCl_3_): 8.10–7.00 (m, 8H), 4.32 (s, 3H), 4.17 (s, 3H), 3.45 (s, 2H). ^13^C NMR (50 MHz, CDCl_3_): 128.6, 126.6, 126.0, 120.2, 111.9, 106.3, 64.0, 32.5, 26.1. Anal. calcd for C_16_H_16_ClIN_2_: C, 48.20; H, 4.05; N, 7.03. Found: C, 42.45; H, 4.90; N, 6.16.

*2-(4-Chlorobenzyl)-1,3-dimethyl-5-trifluoromethyl-1 H-benzimidazol-3-ium iodide (****22****):* Yield: 41%. m.p. 60 °C (Et_2_O an.). ^1^H NMR (200 MHz, CDCl_3_): 8.16–7.08 (m, 7H), 4.25 (s, 3H), 4.18 (s, 3H), 3.58 (s, 2H). ^13^C NMR (50 MHz, CDCl_3_): 153.4, 132.7, 130.4, 128.7, 126.1, 123.2, 113.4, 110.0, 64.1, 33.4, 33.1, 30.9, 28.9. Anal. calcd for C_17_H_15_ClF_3_IN_2_: C, 43.75; H, 3.24; N, 6.00. Found: C, 43.92; H, 3.49; N, 6.00.

*2-(4-Chlorophenyl)-3-methyl-1-{[5-methylammonio-(1 S,9aR)-octahydroquinolizin-1-yl]- methyl}-5-trifluoromethyl-1 H-benzimidazol-3-ium diiodide (****39****):* Yield: 35%. m.p. 165–169 °C (Et_2_O an.). ^1^H NMR (200 MHz, DMSO): 8.75 (s, 1 arom. H), 8.56 (d, *J* = 9.0, 1H), 8.07 (d, *J* = 8.5, 2H), 7.91 (d, *J* = 8.5, 2H), 7.26 (s, 1H), 3.93 (s, 3H), 3.16 (s, 3H), 3.11–2.88 (m, 2H), 2.78–2.63 (m, 2H), 2.17–1.05 (m, 14H). ^13^C NMR (DMSO): 151.5, 138.2, 133.1, 132.3, 131.5, 130.0, 127.2, 123.0, 119.0, 115.1, 66.0, 64.5, 50.3, 49.1, 47.2, 33.4, 33.0, 20.6, 19.1, 18.7, 18.1. Anal. calcd for C_26_H_31_ClF_3_I_2_N_3_: C, 40.52; H, 4.97; N 5.47. Found: C, 40.67; H, 4.59; N 5.47.

### Evaluation of anti-leishmanial activity

- Promastigote stage of *L. infantum* strain MHOM/TN/80/IPT1 (kindly provided by Dr M. Gramiccia, ISS, Roma) and *L. tropica* (MHOM/IT/2012/ISS3130) were cultured in RPMI 1640 medium (EuroClone) supplemented with 10% heat-inactivated fetal calf serum (EuroClone), 20 mM Hepes, and 2 mM *L*-glutamine at 24 °C.

To estimate the 50% inhibitory concentration (IC_50_), the MTT (3-[4.5-dimethylthiazol-2-yl]-2.5-diphenyltetrazolium bromide) method was used^31^^,^^32^. Compounds were dissolved in DMSO and then diluted with medium to achieve the required concentrations. Drugs were placed in 96 wells round-bottom microplates and seven serial dilutions made. Amphotericin B or miltefosine were used as reference anti-*Leishmania* drugs. Parasites were diluted in complete medium to 5 × 10^6^ parasites/mL and 100 μL of the suspension was seeded into the plates, incubated at 24 °C for 72 h and then 20 µL of MTT solution (5 mg/mL) was added into each well for 3 h. The plates were then centrifuged at 1000 × *g* for 8 min at r.t., the supernatants discarded and the resulting pellets dissolved in 100 µL of lysing buffer consisting of 20% (w/v) of a solution of SDS (Sigma), 40% of DMF (Merck) in H_2_O. The absorbance was measured spectrophotometrically at a test wavelength of 550 nm and a reference wavelength of 650 nm. The results are expressed as IC_50_ which is the dose of compound necessary to inhibit parasite growth by 50%; each IC_50_ value is the mean of separate experiments performed in duplicate.

(b) *In vitro* intracellular amastigote susceptibility assays. THP-1 cells (human acute monocytic leukaemia cell line) were maintained in RPMI supplemented with 10% FBS, 50 μM 2-mercaptoethanol, 20 mM Hepes, 2 mM glutamine, at 37 °C in 5% CO_2_. For *Leishmania* infections, THP-1 cells were plated at 5 × 10^5^ cells/mL in 16-chamber Lab-Tek culture slides (Nunc) and treated with 0.1 μM phorbol myristate acetate (PMA, Sigma) for 48 h to achieve differentiation into macrophages. Cells were washed and infected with metacyclic *L. infantum* promastigotes at a macrophage/promastigote ratio of 1/10 for 24 h. Cell monolayers were then washed and incubated with compounds for 72 h. Slides were fixed with methanol and stained with Giemsa. The percentage of infected macrophages in treated and non-treated cells was determined by light microscopy.

### Cell cytotoxicity assays

(a) The long-term human microvascular endothelial cell line (HMEC-1) was maintained in MCDB 131 medium (Invitrogen, Milan, Italy) supplemented with 10% fetal calf serum (HyClone, Celbio, Milan, Italy), 10 ng/mL of epidermal growth factor (Chemicon), 1 µg/mL of hydrocortisone, 2 mM glutamine, 100 U/mL of penicillin, 100 l g/mL of streptomycin and 20 mM Hepes buffer (EuroClone). Unless stated otherwise, all reagents were from Sigma Italia, Milan, Italy. For the cytotoxicity assays, cells were treated with serial dilutions of test compounds and cell proliferation evaluated using the MTT assay already described^33^. The results are expressed as IC_50_, which is the dose of compound necessary to inhibit cell growth by 50%.

(b) Vero-76 cells were seeded at an initial density of 4 × 10^5^ cells/mL in 24-well plates, in culture medium (Dulbecco’s Modified Eagle Medium (D-MEM) with l-glutamine, supplemented with foetal bovine serum (FBS), 0.025 g/L kanamycin). Cell cultures were then incubated at 37 °C in a humidified, 5% CO_2_ atmosphere in the absence or presence of serial dilutions of test compounds. Cell viability was determined after 48–96 h at 37 °C by the Crystal violet staining method.

The results are expressed as CC_50_, which is the concentration of compound necessary to inhibit cell growth by 50%. Each CC_50_ value is the mean and standard deviation of at least three separate experiments performed in duplicate.

## Results and discussion

### Synthesis

**Scheme 1.**
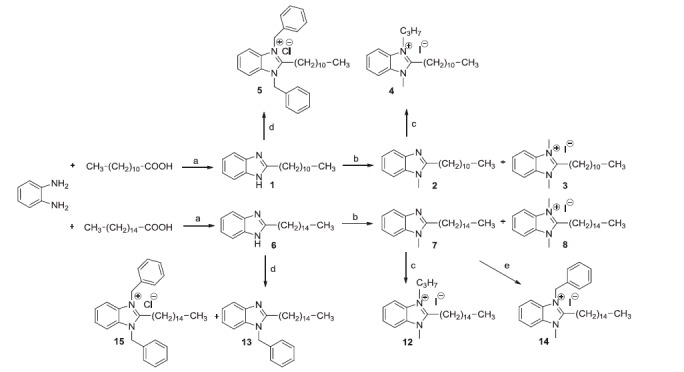
Reagents and conditions: (a) 145 °C, N_2_, 24 h; (b) CH_3_I, THF, K_2_CO_3_, 40 °C, 76 h; (c) C_3_H_7_I, THF, 24–60 h; (d) C_6_H_5_–CH_2_–Cl, THF, K_2_CO_3_, N_2_, reflux, 60 h; (e) C_6_H_5_–CH_2_–Cl, THF, N_2_, reflux, 80 h.

The 1-unsubstituted 2-alkylbenzimidazoles were prepared either by dry heating at 145 °C of a mixture of 1,2-phenylenediamine with the suitable acid (**1** and **6**), or by treating the diamine with valproyl chloride, in dioxane solution, followed by the action of ethereal boron trifluoride (**18**) (Schemes 1 and 4). The last method, in contrast with the indication of Tandon and Kumar^34^, gave only a modest yield of benzimidazole, being prevailing the formation of the N,N’-divalproyl-1,2-phenylendiamine (**49**). Compounds **1** and **6** were already described (see Materials and methods).

The treatment of the 1-unsubstituted benzimidazoles with excess of methyl iodide, in the presence of anhydrous K_2_CO_3_, gave place to mixtures of 1-methyl-2-substituted benzimidazoles (**2**, **7** and **19**) and 1,3-dimethyl-2-substituted benzimidazolium iodides (**3**, **8** and **20**) (Schemes 1 and 4). The dimethylated compounds were easily isolated being insoluble in dry ether, while the monomethylated compounds were separated from the N-unsubstituted benzimidazoles by CC on silica, eluting with CH_2_Cl_2_. Similarly, by treating compounds **1** and **6** with an excess of benzyl chloride the 1,3-dibenzyl benzimidazolium chlorides **5** and **15** were obtained, but the mono-benzylated compound (**13**) was isolated only in the case of **6** (Scheme 1). Compounds **2**, **3** and **8** were already described (see Materials and methods).

An attempt to improve the yield of 1-methyl-2-pentadecyl benzimidazole (**7**) by reacting directly the palmitic acid with N-methyl-1,2-phenylendiamine gave disappointing result (yield 17%).

**Scheme 2.**
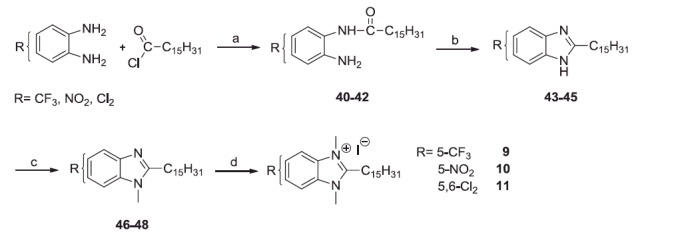
Reagents and conditions: (a) THF, N_2_, Hünig base (2 equiv), r.t., 24 h; (b) HCl 4 N, reflux, 4 h; (c) CH_3_I, THF, Cs_2_CO_3,_ 60 °C_,_ 6–8 h; (d) CH_3_I excess, r.t., 24 h.

To obtain the 5-substituted compounds **9**–**11**, the 4-substituted or 4,5-disubstituted-1,2-phenylenediamines were mono-acylated with palmitoyl chloride and the monoamides **40**–**42** were cyclised by the action of 4 N HCl. The benzimidazoles **43**–**45** were methylated with methyl iodide in the presence of Cs_2_CO_3_ (**46**–**48**) and, finally, quaternised at r.t. with excess of methyl iodide (Scheme 2). The intermediates **40**, **41**, **46** and **47** (Scheme 2) could be a mixture of two regioisomers, however we did not succeed in separating them, but it is not important for the structures of the final compounds **9**–**11**.

**Scheme 3.**
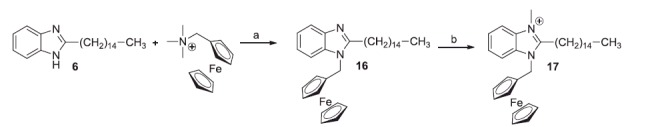
Reagents and conditions: (a) CH_3_CN/THF, K_2_CO_3_, N_2_, r.t., 18 h; (b) CH_3_I, dry Et_2_O, 40 °C, 80 h.

The mono-methylated benzimidazoles **2** and **7** were converted into the quaternary salts **4**, **12** and **14** (Scheme 1), by heating them with propyl iodide or with benzyl chloride for the latter. As suggested by Howarth and Hanlon^35^ for analogous compounds, by treating the 2-pentadecyl benzimidazole with (ferrocenylmethyl)trimethyl ammonium iodide at r.t., the 1-ferrocenylmethylbenzimidazole **16** was obtained in high yield, the latter was then quaternised with methyl iodide to **17** (Scheme 3).

**Scheme 4.**
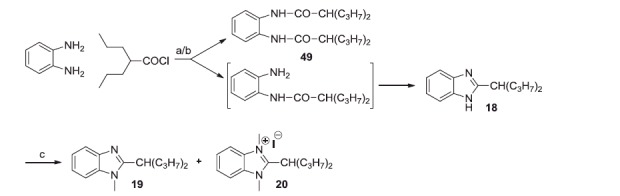
Reagents and conditions: (a) dioxane, N_2_, r.t., 15 h; (b) BF_3_*Et_2_O, reflux, 12 h; (c) CH_3_I, THF, K_2_CO_3_, 50 °C, 26 h.

**Scheme 5.**
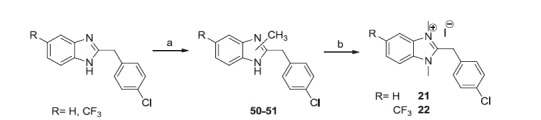
Reagents and conditions: (a) CH_3_I, THF, Cs_2_CO_3_, 60 °C, 6 h; (b) CH_3_I excess, r.t., 24 h.

Finally, by treating the 2-(4-chlorobenzyl)benzimidazole^36^ and 2-(4-chlorobenzyl)-5-trifluoromethylbenzimidazole^37^ with methyl iodide in the presence of Cs_2_CO_3_, the corresponding 1-methylbenzimidazoles were obtained, that with excess of methyl iodide gave the quaternary salts **21** and **22** (Scheme 5).

**Scheme 6.**
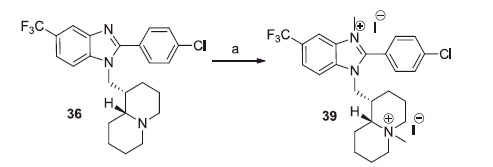
Reagents and conditions: (a) CH_3_I excess, r.t., 24 h.

All but one (**39**) of the benzimidazole derivatives bearing a basic side chain were already described by some of us: **24**, **28** and **32**–**34**^21a^; **23**, **25** and **29–31**^21b^; **26** and **27**^24c^; **35**, **36** and **38**^21c^; **37**^25b^. The novel bisquaternary salt **39** was obtained by treating with methyl iodide the previously described benzimidazole derivative **36**^21c^ (Scheme 6). Attempts of selective quaternisation of quinolizidine nitrogen were unsuccessful.

### Antileishmanial activity

With the exception of compound **2**, all the (**38**) compounds of Figures 4 and 5 were tested *in vitro* against promastigotes of *L. tropica*, while 33 of them were also tested against *L. infantum*, using the MTT assay^31^^,^^32^. Results are expressed as IC_50_ ± SD (µM) and reported in Table 1, together with the corresponding selectivity indexes (ratio of IC_50_ versus human microvascular endothelial cell line (HMEC-1), or monkey kidney cells (Vero76), and IC_50_ of compounds versus the two *Leishmania* species.

**Table 1.** *In vitro* data on antileishmanial activity against *L. tropica* and *L. infantum* promastigotes and cytotoxicity on the human endothelial cell line (HMEC-1) and/or monkey kidney cell (Vero-76) of benzimidazole derivatives **1** and **3**–**39**.

Compd.	IC_50_ (µM)^a^*L. tropica*	IC_50_ amph. B ×100/IC_50_ compd.^b^	Ratio^c^ IC_50_ miltef./IC_50_ compd.	IC_50_ (µM)^a^*L. infantum*	IC_50_ amph. B ×100/IC_50_ compd.^b^	Ratio^c^ IC_50_ miltef./IC_50_ compd.	IC_50_ (µM) HMEC-1 and or Vero76^d^	SI^e^*L. tropica*	SI^e^*L. infantum*
**1**	>16.20*	/	/	nt	/	/	51.00 ± 7.5	/	/
**3**	1.68*	4.9	25.8	0.28 ± 0.07	55.7	111.6	2.64 ± 0.49	1.57	9.43
**4**	0.46*	17.9	94.0	0.27 ± 0.01	57.8	115.8	2.01 ± 0.24/2.6 ± 0.3	4.37/5.65	7.44/9.63
**5**	0.78*	10.5	55.5	0.61 ± 0.05	25.6	51.3	1.38 ± 0.17	1.77	2.26
**6**	5.05 ± 0.01	1.6	8.6	10.09 ± 4.9	0.93	3.1	>37	>27.1	>13.6
**7**	>58.0	/	/	>58.0	/	/	nt	/	/
**8**	0.19 ± 0.06	43.5	227.7	0.34 ± 0.12	27.8	91.9	0.78 ± 0.06/5.8 ± 0.3	4.10/30.5	2.29/17.1
**9**	0.87 ± 0.16	11.9	49.7	1.32 ± 0.30	8.9	23.7	1.86 ± 0.34	2.14	1.41
**10**	1.40 ± 0.58	7.35	30.9	0.96 ± 0.17	12.3	32.6	2.11 ± 0.45	1.51	2.20
**11**	1.06 ± 0.54	9.1	40.8	0.84 ± 0.13	14.1	37.2	1.68 ± 0.75	1.58	2.00
**12**	0.51*	16.1	84.8	0.42 ± 0.14	37.1	74.4	0.73 ± 0.37	1.43	1.74
**13**	>11.90	/	/	>11.90	/	/	>47.0	/	/
**14**	0.49*	16.7	88.3	0.64 ± 0.01	24.4	48.8	0.91 ± 0.19	1.86	1.42
**15**	1.61 ± 0.15	5.1	26.9	3.26 ± 0.80	2.9	9.6	0.94 ± 0.17	0.60	0.29
**16**	>35.0	/	/	nt	/	/	>35.0	/	/
**17**	3.56 ± 0.84	3.34	12.2	nt	/	/	1.26 ± 0.24	0.35	/
**18**	11.08 ± 2.73	1.07	3.9	>39.6	/	/	65.10 ± 12.21	5.87	/
**19**	30.02 ± 10.27	0.40	1.4	nt	/	/	>74.0	>2.43	/
**20**	>73.0	/	/	nt	/	/	>73.0	/	/
**21**	>50.0	/	/	>50.0	/	/	nt	/	/
**22**	33.93 ± 12.62	0.30	1.3	16.74 ± 7.07	0.70	1.9	nt	/	/
**23**	47.26 ± 13.25	0.36	0.9	>56.0	/	/	nt	/	/
**24**	15.04 ± 1.03	1.18	2.9	20.89 ± 7.57	1.0	1.5	>100	>6.65	>4.79
**25**	>51.0	/	/	>51.0	/	/	nt	/	/
**26**	29.64 ± 0.30	0.60	1.5	>55.0	/	/	>100	>3.37	/
**27**	21.09 ± 5.84	0.84	2.1	32.68 ± 3.92	0.64	1.0	>100	>4.74	>3
**28**	3.70 ± 1.19	4.8	11.7	4.76 ± 1.60	4.4	6.6	16.95/>100	4.58/>27	3.56/>21
**29**	23.71 ± 8.54	0.75	1.8	28.51 ± 8.66	0.73	1.1	nt	/	/
**30**	12.13 ± 4.06	1.46	3.6	15.87 ± 3.21	1.32	2.0	>100	>8.24	>6.30
**31**	23.89 ± 7.78	0.74	1.8	28.52 ± 4.47	0.73	1.1	nt	/	/
**32**	7.22 ± 3.13	1.43	6.0	9.55 ± 3.11	1.24	3.3	50	6.93	5.23
**33**	3.92 ± 1.43	2.63	11.0	6.82 ± 1.25	1.73	4.6	68*	17.35	9.97
**34**	3.44 ± 1.43	2.99	12.6	6.68 ± 1.74	1.77	4.7	49*	14.24	7.33
**35**	7.31 ± 2.75	2.42	5.9	12.09 ± 1.82	1.73	2.6	90*	12.3	7.44
**36**	12.61 ± 4.54	0.69	3.4	12.47 ± 3.51	0.95	2.5	24*	1.90	1.92
**37**	13.53 ± 2.21	1.31	3.2	17.36 ± 1.49	1.20	1.8	78*	5.76	4.49
**38**	6.54 ± 0.18	2.71	6.6	11.23 ± 1.33	1.86	2.8	75*	11.47	6.68
**39**	>27.0	/	/	>27.0	/	/	nt	/	/
Amph. B	0.113 ± 0.03^f^	100		0.135 ± 0.03^f^	100		25.7 ± 1.90^g^	227.4	190.4
Miltefosine	43.26 ± 11.36	0.26	1.0	31.26 ± 10.43	0.27	1.0	99.8*^h^	2.3	3.2

a The results are expressed as IC_50_ ± SD of at least three different experiments performed in duplicate or triplicate, with the exception of the starred* values that are the means of two experiments performed in duplicate.

b Ratios between the IC_50_ of amphotericin B × 100 and IC_50_ of each compound against *L. tropica* or *L. infantum*, calculated for each experiment. The IC_50_ values of amphotericin B ranged from 0.082 to 0.177 µM for *L. tropica*, and from 0.094 to 0.209 µM for *L. infantum*.

c Ratios between the IC_50_ of miltefosine and that of each compound against *L. tropica* or *L. infantum*.

d The cytotoxicity was assayed *in vitro* on the human microvascular endothelial cell line (HMEC-1) for compounds **1**–**20** and **28**, and on monkey kidney (Vero76) cells for compounds **4**, **8**, **23**, **26**–**28**, **30** and **32–39**.

e Selectivity index: IC_50_ HMEC-1 or Vero76/IC_50_ for the two species of *Leishmania*.

f Mean values from many different experiments; range 0.082–0.177 µM for *L. tropica*, and 0.094–0.209 µM for *L. infantum*.

g Cytotoxicity of amphotericin B on HMEC-1 cells.

h Cytotoxicity of miltefosine on HMEC-1 cells.

The results collected in Table 1 show that most of the tested compounds were active against *L. tropica* (30 over 38) and *L. infantum* (25 over 33). Among the compounds considered inactive (**1**, **7**, **13**, **16**, **20**, **21**, **25** and **39**), two (**1** and **13**) were tested only at concentrations up to 16 and 12 μM, respectively, and it is not excluded that they could exhibit some activity at higher concentrations. The active compounds resulted less potent than the reference drug amphotericin B, reaching, at the best, the 43% of its potency versus *L. tropica* (cpd **8**) and the 58% versus *L. infantum* (cpd **4**), respectively. However, comparing the tested compounds with miltefosine, another commonly used drug, they frequently resulted many fold (up to 228-fold) more potent. It is worth noting that in our experimental conditions miltefosine displayed an IC_50_ value versus the promastigote stage of *L. infantum* (31.26 μM) quite higher than the corresponding values found in the literature (15.0 μM^11a^; 16.7 μM^6c^; 19.6 μM^13b^), while no data are available in the literature for miltefosine activity versus *L. tropica* to compare with our results (43 μM). Indeed, substantial variability has been observed for miltefosine susceptibility of several other *Leishmania* species^38^. Anyhow, even taking into account the lowest IC_50_ value (15 μM) aforementioned, most of the tested compounds remain many-fold (up to 55- and 43-fold for compounds **4** and **8**, respectively) more potent than miltefosine against *L. infantum*.

*L. infantum* was commonly (with the exception of compounds **4**, **5**, **10–12** and **22**) less sensitive than *L. tropica*, which in two cases (compounds **23** and **26**) was the only affected species.

Activity was largely present in both subsets of compounds, but the higher potencies (IC_50_≤ 5 µM) were mainly found among the 2-undecyl- and 2-pentadecylbenzimidazole derivatives, in which subset the activity was particularly high (IC_50_ minor or around 1 µM) when the benzimidazole ring was quaternised (compounds **3**–**5**, **8**–**12** and **14**).

**Figure 6.**
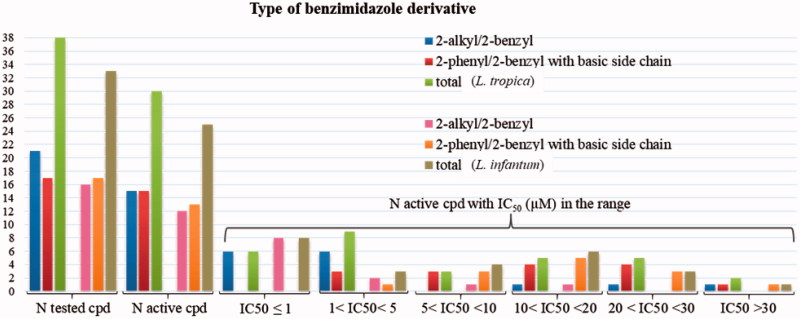
Number of compounds inhibiting the growth of *L. tropica* and *L. infantum* promastigotes and range of their IC_50_ (µM).

Considering the quite different structural features that characterise the two subsets of compounds, the structure–activity relationships will be discussed separately for each subset. The distribution of activity among the two subsets is illustrated in Figure 6.

Regarding the subset of benzimidazole derivatives bearing in position 2 an aliphatic chain, it is observed that the 1-unsubstituted-2-alkylbenzimidazoles (**1**, **6** and **18**) were not only either inactive or only moderately active, but also the least toxic versus HMEC-1 cells. The introduction in position 1 of a methyl, benzyl and ferrocenylmethyl residue abolished (**7**, **13** and **16**) or reduced (**19**) the activity. However generating a fixed positive charge on the benzimidazole ring of the aforementioned compounds, by treating them with methyl or propyl iodide or benzyl chloride, a striking increase of activity was observed, obtaining compounds with IC_50_ in submicromolar (**4**, **5**, **8**, **12** and **14**) or low micromolar range (**3**, **9**–**11**, **15** and **17**). Somewhat unexpected was the lack of activity observed for the quaternised compound **20** (1,3-dimethyl-2-(4-heptyl)benzimidazolium iodide), which was inactive even at a concentration up to 73 µM. Commonly, the quaternisation increased both the activity and the cytotoxicity, while quaternising compound **19** to **20**, its activity was abolished leaving unchanged the low cytotoxicity.

In this subset of benzimidazole derivatives, compounds **4** and **8** were the most potent versus *L. infantum* and *L. tropica*, respectively, with IC_50_= 0.27 and 0.19 µM corresponding to the 58 and 28% of amphotericin B potency with respect to the two *Leishmania* species. In comparison to miltefosine, compound **4** was 116-fold more effective versus *L. infantum*, whilst compound **8** was 228 more potent versus *L. tropica*. The introduction of electron-withdrawing substituents on the benzimidazole ring reduced the activity (compare **8** with **9**–**11**), but the activity-lowering effect was stronger versus *L. tropica* than versus *L. infantum*. However, comparing the couple of compounds **8**–**9** to **21**–**22**, where the pentadecyl chain is replaced by a 4-chlorobenzyl moiety, it is observed that the introduction of a 5-trifluoromethyl group had a positive effect on the activity. Also in this case the activity on *L. infantum* was higher than on *L. tropica*. The toxicity of **4** and **8** (and similar compounds) versus the HMEC cells was not negligible, with selectivity index (SI) in the range 2.3–7.4, that, however, were better than the corresponding SI of miltefosine (2.0 and 3.2). Indeed, the HMEC cells are particularly sensitive to most kinds of chemicals, thus the best compounds **4** and **8** were also tested for toxicity against Monkey kidney Vero76 cells, sharing a quite more valuable SI value. Interestingly, the 1-unsubstituted benzimidazole **6**, even displaying a moderate activity, exhibited a very valuable SI versus the sensitive HMEC cells (SI> 37 and >13). Thus, compounds **4**, **6** and **8** represent interesting hit compounds for developing better anti-leishmania agents by increasing activity or reducing toxicity through further chemical manipulation (chain length, chain branching and unsaturation, number and nature of substituents on the benzimidazole and eventual benzyl group).

Compound **8** and its analogues (**3**–**5**, **9**–**12**, **14**, **15** and **17**) may display their activity (as well as their toxicity) acting as *cationic* surfactants able to modify, like miltefosine^38^, the cell membrane permeability; moreover, once inside the cell, they may activate several stress pathways, inhibit fatty acids and sterol biosynthesis, and/or cytochrome-C oxidase and other targets. Moreover, it is known that quaternary ammonium compounds are able to impair the uptake of choline^39^, required for the synthesis of parasite membrane phospholipids, but also to inhibit the 3-fold methylation of phosphatidyl ethanolamine that represents the primary route to the *Leishmania* phosphatidyl choline^40^. It is worth noting that sodium 2-pentadecylbenzimidazole-5-carboxylate (M&B35347B) besides acting as *anionic* surfactant, is an inhibitor of acetyl-CoA carboxylase able to derange fatty acid and cholesterol biosynthetic pathways^41^.

Concerning the subset of 2-phenyl- and 2-benzylbenzimidazoles, the compounds bearing an open-chain basic head were only moderately active (**24**, **26** and **27**) or inactive (**23** and **25**), while those bearing a lupinyl residue were all, but one (**39**), endowed with valuable activity. Among the 1-lupinylbenzimidazole the activity was influenced by the substituents in 2 and 5 positions. The 5-acetyl derivatives were less potent than the corresponding 5-trifluoromethyl- and 5-nitro derivatives (compare **28**–**34**, particularly **28**, **30** and **33**). The negative effect of the 5-acetyl group was also evident among the 1-dialkylaminoalkyl derivatives **23–27**.

The higher potency of compound **28** in comparison to **36** suggests that the 2-benzylbenzimidazoles may be more potent than the corresponding 2-phenyl analogues, and indeed, excluding from comparison compounds **29**–**31** for the presence of the acetyl group (negatively affecting the activity), the 2-benzyl-1-lupinylbenzimidazole were, on average, more potent than the 2-phenyl-1-lupinyl derivatives.

With this kind of compounds we did not succeed to quaternise the lupinyl moiety without affecting also the benzimidazole ring and it was observed that the double quaternisation produced the loss of activity (compare compounds **36** and **39**).

In this subset of 2-arylbenzimidazoles, compound **28** appears as the most interesting because resulted 12-/7-fold more potent than miltefosine and did not manifest any discernible cytotoxicity on Vero cells (CC_50_> 100 µM and SI >27 and >21 versus *L. tropica* and *L. infantum*, respectively), while the toxicity on HMEC-1 cells was only moderate (SI= 4.58 and 3.56 versus the two *Leishmania* species). It is worth noting that compound **28** was already shown to possess antiproliferative activity, with GI_50_< 5 µM, against 24 human cancer cell lines, among which the renal cancer cell line UO31 was particularly sensitive (GI_50_= 0.019 µM^25b^). Moreover, the same compound displayed moderate antiviral activity against Coxsackie virus B5 (CVB-5) and respiratory syncytial virus (RSV) with EC_50_ 13 and 15 µM, respectively^24c^. Also compounds **33** and **34** displayed good level of antileishmanial activity associate with modest toxicity on Vero76 cells and represent, together with **28**, interesting hit compounds.

Possessing a basic side chain, compounds **23**–**39** might, like sitamaquine^3b^^,^^4d^, anchor to the anionic phospholipidic components of *Leishmania* cell membrane, disrupting its function.

Eventually, they could permeate the cell and accumulate into cytosolic acidic compartments. Once inside the cells, the benzimidazole derivatives might inhibit some of the enzymes that are essential for *Leishmania* survival and proliferation and are absent from their mammalian host^42^, like those involved in the biosynthesis of membrane ergosterol and the 24-alkylsterols^3b^^,^^43^ or the zinc metalloprotease (leishmanolysin)^18^^,^^44^, playing crucial roles in the *Leishmania* parasite physiology and in host-parasite interaction.

Some benzimidazole derivatives bearing a basic side chain have, already, been shown to somewhat affect sterol biosynthesis, like 2-[(4-diethylaminoethoxy)phenyl]benzimidazole that blocks the reduction of 7-dehydrocholesterol to cholesterol^45^ and 2-(4-chlorobenzyl)-1-(3-diethylaminopropyl)-5-trifluoromethylbenzimidazole^21^ (structurally close to compounds **23–25**) that, at 50 mg/kg *p.os*, reduced significantly (>15%) the serum cholesterol concentration in hypercholesterolemic mice. The mechanism of action of these two kinds of benzimidazole derivatives was not further investigated, and the possibility of their interference in parasite ergosterol biosynthesis may be only conjectural.

On the other hand, some 2-aryl-5-substituted benzimidazoles, devoid of basic chain, have been shown to inhibit the stearoyl coenzyme A desaturase (SCD1), blocking the formation of oleic and palmitoleic triglycerides, cholesterol esters and phospholipids^46^. The SCD1, besides being investigated for the treatment of dislipidemic diseases and body weight control, has been found to participate, together with other desaturase enzymes, in the *de novo* synthesis of mono- and poly-unsaturated fatty acids (C18–C22 PUFA) of parasitic membrane. These biosynthetic pathways play a crucial role for parasitic viability at different life cycle stages^47^. Some other 2-arylbenzimidazoles, still lacking basic side chain (Figure 3, central row), have been shown to exhibit leishmanicidal effect and to dock successfully in the binding pocket of the promastigote surface protease (leishmanolysin, GP63 protein), which contributes to parasite virulence^18^. Of course, for the discussed compounds, other, even multiple, mechanisms of action, not yet identified, may take place.

Finally, for a better insight of the real value of the studied compounds as antileishmanial agents, compounds **8** and **28**, representative of the two subsets of benzimidazole derivatives that display the highest activity against the promastigote stage, were tested against the intramacrophagic amastigote stage of *L. infantum*. Compound **8** exhibited an IC_50_= 0.313 μM, with a 3.35-fold increased potency with respect to miltefosine, while compound **28**, at 2 μM concentration (42% of its IC_50_ versus promastigotes) reduced the amastigote infection of THP-1 cells by 33.2% (human acute monocytic leukaemia cell line; IC_50_> 2 μM).

## Conclusions

Two sets of benzimidazole derivatives (38 compounds) were tested *in vitro* for activity against promastigotes of *L. tropica* and *L. infantum*. A first set was formed by 2-(long chain)-alkyl/benzyl benzimidazoles (**1**–**22**), whose heterocyclic head was, in most cases, quaternised to mimic the ammonium head of miltefosine and related analogous anti-leishmanial drugs. The second set was composed of 2-benzyl and 2-phenyl benzimidazoles (**23**–**39**) bearing in position 1 a basic side chain (dialkylaminoalkyl- or lupinyl-).

Most of the tested compounds of both sets resulted active against *L. tropica* (30 over 38) and *L. infantum* (25 over 33) (Figure 6). The IC_50_ values for the quaternised 2-alkylbenzimidazoles were in the low micromolar/submicromolar range. Compound **8** (IC_50_= 0.19 µM and 0.34 µM versus *L. tropica* and *L. infantum*, respectively) resulted 228- and 93-fold more potent than miltefosine, with SI in the range 4.1–2.3 versus HMEC cells, but displaying SI= 30 and 17 versus Vero76 cells. Among the compounds bearing a basic side chain, the 1-lupinyl derivatives were commonly more active than dialkylaminoalkyl ones, and compound **28** [2-(4-chlorobenzyl)-1-lupinyl-5-trifluoromethylbenzimidazole] displayed the highest potency (IC_50_= 3.70 µM and 4.76 µM for the two *Leishmania* species). This compound was just a little less toxic than **8** on HMEC cells (SI= 4.6 and 3.6 versus *L. tropica* and *L. infantum*, respectively), but did not manifest any discernible cytotoxicity against Vero76 cells (CC_50_> 100 µM and SI= 27 and 21 versus the two *Leishmania* species). Therefore, several compounds and particularly the benzimidazoles **8** and **28**, whose activity was confirmed on intramacrophagic amastigote stage of *L. infantum*, represent interesting hit compounds, whose structure can be further variate in order to improve their safety profiles (toxicity/activity ratios).

Based on the chemical features of the relevant compounds, their interaction with the acidic components (mainly the phospholipids) of cell membrane, with consequent disruption of its function, may explain the observed anti-leishmanial activity. The internalisation of compounds and their interaction with different targets inside the cell might also have an important role, but its investigation is beyond the aim of the present preliminary study.
